# Friedlander’s Pneumonia with Recovery

**Published:** 1916-03

**Authors:** 


**Friedlander's Pneumonia With Recovery.** C. H. Dunn and J. Hammond, Interstate Med. Jour., Nov. 1915, report a case in a male aged 14½ months. Clinically, the case was one of left lower lobe pneumonia, with crisis and reinfection located in the left upper lobe. Puncture showed no pleuritic effusion but, the needle entering the lung brought away a small amount of fibrinous exudate from which the bacillus mucosus capsulatus was cultivated. This case is said to be the only one reported in an infant and the only case to recover.

